# Enhanced efficacy of aprepitant-based triple prophylaxis in preventing postoperative nausea and vomiting following metabolic bariatric surgery: a single-center, retrospective cohort study

**DOI:** 10.3389/fmed.2025.1481720

**Published:** 2025-02-17

**Authors:** Xiaodong Shan, Yidi Yang, Xiaoao Xiao, Mingchuang Zhang, Rui Chen, Qingqiu Huang, Yuanqing Gao, Xitai Sun

**Affiliations:** ^1^Department of Pancreatic and Metabolic Surgery, Nanjing Drum Tower Hospital, Affiliated Hospital of Medical School, Nanjing University, Nanjing, China; ^2^Nanjing Drum Tower Hospital, Clinical College of Nanjing Medical University, Nanjing, China; ^3^Key Laboratory of Cardiovascular and Cerebrovascular Medicine, School of Pharmacy, Nanjing Medical University, Nanjing, China; ^4^Department of Pancreatic and Metabolic Surgery, Nanjing Drum Tower Hospital, Clinical College of Nanjing University of Chinese Medicine, Nanjing, China

**Keywords:** obesity, bariatric surgery, postoperative nausea and vomit, antiemetic prophylaxis, multimodal, aprepitant

## Abstract

**Background:**

Metabolic bariatric surgery (MBS) is associated with high risk of postoperative nausea and vomiting (PONV). We aimed to investigate the impact of aprepitant-based triple prophylaxis on PONV after MBS.

**Methods:**

We reviewed a retrospective cohort of patients who underwent primary MBS between December 28, 2023 and May 31, 2024. The eligible patients were divided into two groups based on whether receiving additional oral single 125 mg dose of aprepitant preoperatively to the dual prophylaxis (ondansetron 8 mg and dexamethasone 10 mg). Multivariable and propensity score-adjusted analyses were performed to compare the composite PONV endpoints between the groups.

**Results:**

A total of 207 patients were included in the study. Of these, 129 patients received dual prophylaxis, while the remaining 78 patients received additional single 125 mg dose of aprepitant. Similar to multivariable logistic regression analysis, propensity-adjusted logistic regression analysis revealed that the aprepitant-based triple prophylaxis group had a significantly higher complete response rate (82.1% vs. 24.0%, adjusted OR 10.312, 95% CI 4.186–25.399, *p* < 0.001), a lower incidence of PONV (59.0% vs. 85.3%, adjusted OR 0.287, 95% CI 0.125–0.663, *p* = 0.004), and required fewer rescue antiemetics (7.7% vs. 37.2%, adjusted OR 0.155, 95% CI 0.052–0.457, *p* < 0.001) compared to the dual prophylaxis group. Propensity score-adjusted analysis demonstrated that the addition of aprepitant to dual prophylaxis significantly reduced the incidence of PONV, vomiting frequency, and both the Nausea VAS and Nausea Subscale scores (all *p* < 0.05).

**Conclusion:**

Our findings indicate that the addition of a single preoperative dose of aprepitant to a dual antiemetic prophylaxis of dexamethasone and ondansetron might be associated with a further improve outcomes related to composite PONV endpoints in patients undergoing metabolic bariatric surgery.

## 1 Introduction

Postoperative nausea and vomiting (PONV) is one of the most common perioperative complications, with an incidence of approximately 40% in the general surgical population. This incidence is even higher in patients undergoing metabolic bariatric surgery (MBS), reaching up to 65% (1), and in those undergoing laparoscopic sleeve gastrectomy (LSG), where it can be as high as 77.4 to 91.4% (2–4). PONV has been strongly associated with increased risks of postoperative bleeding, dehydration, electrolyte imbalance, acute kidney injury, prolonged length of stay, pulmonary aspiration and decreased patient satisfaction (5–8). The increased incidence of PONV can significantly limit early mobilization, thus increasing the risk of postoperative pulmonary complications and deep vein thrombosis. Effective prevention and treatment of PONV can improve patient outcomes and satisfaction. Currently, the majority of metabolic and bariatric surgery centers, including our institution, have adopted institutional Enhanced Recovery after Surgery (ERAS) protocols for their surgical patients. Recognizing the high incidence and low control rates of PONV in MBS, the American Society for Metabolic and Bariatric Surgery and the International Society for the Perioperative Care of Patients with Obesity have jointly issued a position statement recommending the use of multimodal antiemetic regimens rather than single-agent therapy, with at least two to three antiemetics from different pharmacological classes for prophylaxis (9). The guidelines also encourage further clinical research into multimodal prophylactic antiemetic regimens. However, even with the current standard of dual antiemetic prophylaxis, the risk of PONV after MBS remains nearly 60% (9).

Aprepitant, a selective neurokinin-1 (NK-1) receptor antagonist with a half-life of 9–13 h, has been shown to effectively reduce emesis induced by opioids, chemotherapy, and surgical anesthesia (10). It has been approved by the U.S. Food and Drug Administration for the prevention of chemotherapy-induced and postoperative nausea and vomiting (11, 12). However, there is limited evidence regarding the role of aprepitant within multimodal antiemetic prophylaxis protocols specifically for bariatric surgery patients, particularly in combination with existing dual antiemetic regimens. Addressing this gap, the present study aimed to test the hypothesis that adding a single preoperative dose of aprepitant to a dual antiemetic prophylactic regimen further improves outcomes related to composite PONV endpoints in patients undergoing MBS. By building on the current literature and exploring more effective antiemetic combinations, this study seeks to optimize PONV management strategies in this high-risk surgical population.

## 2 Materials and methods

### 2.1 Study design and patients

This study was a retrospective cohort study. We reviewed the patients who underwent MBS at the Nanjing Drum Tower Hospital between December 28, 2023 and May 31, 2024. The study cohort was divided into two groups: dual prophylaxis group (Ondansetron 8 mg + Dexamethasone 10 mg) and triple prophylaxis group (Ondansetron 8 mg + Dexamethasone 10 mg + Aprepitant 125 mg).

#### 2.1.1 Inclusion criteria

Patients who underwent primary MBS at Nanjing Drum Tower Hospital between December 28, 2023, and May 31, 2024, were enrolled in this study. All participants were required to have pre-operative liver and kidney function within normal limits, as defined by aspartate aminotransferase (AST) and alanine aminotransferase (ALT) levels ≤3.0 times the upper limit of normal (ULN), bilirubin levels ≤ ULN, and creatinine levels ≤1.5 times ULN.

#### 2.1.2 Exclusion criteria

Patients with incomplete data on PONV were excluded from the study. Additionally, individuals with a history of prior bariatric surgery or those undergoing concurrent procedures, such as cholecystectomy, in combination with the primary MBS were excluded. Patients who reported any symptoms of nausea or vomiting, or who had used antiemetics within 1 week prior to surgery were also excluded from the study.

### 2.2 Data collection

Demographic and clinical data (age, sex, body mass index (BMI), smoking history, history of motion sickness, American Society of Anesthesiologists physical status (ASA-PS) score, obesity-related comorbidities, etc.), anesthesia characteristics (anesthesia duration, opioid dosage, intraoperative total intravenous fluid volume (TIV), urine volume), surgical characteristics (surgery type, surgical duration, bleeding volume), composite PONV endpoints (frequency of vomiting or retching, the simplified PONV impact scale score, nausea visual analog scale (VAS) scores and use of rescue antiemetics), and other eligibility criteria data were retrospectively collected from electronic medical records and a bariatric surgery database. In this study, a positive smoking history was defined as having a prior habit of smoking, with cessation occurring before admission. Regarding the handling of missing data, the majority of the data in this study were routinely collected preoperatively and intraoperatively as part of MBS protocols. As such, there were no missing data for preoperative and intraoperative variables. However, in cases where postoperative PONV-related outcome data were missing, these patients were excluded from the study in accordance with the pre-specified exclusion criteria.

### 2.3 Surgical techniques of metabolic bariatric surgery

#### 2.3.1 Laparoscopic sleeve gastrectomy

The patient is placed in the reverse Trendelenburg position with a flat abdomen. A four-port technique is utilized. The omental tissue and vessels are dissected closely along the greater curvature of the stomach, extending distally to 2–4 cm from the pylorus and proximally to the left side of the gastroesophageal junction. The left diaphragmatic angle and His angle are fully exposed. The posterior wall of the stomach is mobilized, and a 36 Fr bougie is placed orally. Guided by the bougie, a linear cutting and stapling device is used to resect the stomach along the greater curvature, from 2 to 4 cm proximal to the pylorus to the 1.5 cm from the His angle. The staple line was reinforced with an additional layer of 3–0 absorbable sutures using a Lembert technique. The final sleeve stomach volume is approximately 60–100 mL.

#### 2.3.2 Single anastomosis sleeve jejunal bypass or single anastomosis sleeve ileal bypass

The procedure utilizes a five-port technique. LSG is performed as described previously. Following LSG, a side-to-side anastomosis is created between the proximal or distal third of the small intestine and the sleeve stomach via a single anastomosis.

All of the aforementioned procedures were performed laparoscopically by the same experienced MBS team.

### 2.4 Standardized anesthesia and analgesia

Anesthetic management adhered to a standardized clinical protocol. Induction of anesthesia was achieved using propofol, vecuronium, and midazolam. Anesthesia was maintained with propofol, atracurium, and dexmedetomidine. Standard intraoperative analgesia was administered with remifentanil, sufentanil, or fentanyl, while postoperative analgesia was managed with flurbiprofen, with opioid analgesics avoided. All patients were managed with total intravenous anesthesia (TIVA) whenever possible, with inhalational anesthesia avoided whenever feasible.

### 2.5 Prophylaxis, rescue, and assessment of PONV

Prophylactic antiemetic regimen: All patients received a dual antiemetic prophylaxis consisting of ondansetron (8 mg) and dexamethasone (10 mg). Specifically, patients were administered intravenous dexamethasone 10 mg at the start of the surgery and intravenous ondansetron 8 mg 30 min before the end of the surgery. In the triple prophylaxis group, patients received the same ondansetron and dexamethasone regimen, with an additional oral dose of aprepitant 125 mg administered 2 h prior to MBS to prevent PONV. Considering the high incidence of PONV after bariatric surgery, aprepitant was used indiscriminately for all bariatric surgery patients over a certain period within the study period, without selecting specific treatment targets. Rescue antiemetic regimen: Based on the severity of PONV, single-dose rescue antiemesis was administered intravenously with metoclopramide 10 mg or ondansetron 4, or intramuscular with promethazine 12.5 mg. If there was no response to the initial dose, a second dose was provided. All surgical patients were routinely assessed for PONV, and data on vomiting episodes, rescue medication use, Simplified PONV Impact Scale scores, and nausea VAS scores were recorded in the PONV database within the metabolic bariatric surgery sub-database.

### 2.6 Endpoint assessment

The occurrence of PONV was recorded and assessed by resident physicians, nurses, and other healthcare professionals for each patient. The primary endpoint was the complete response (CR) rate, defined as the absence of vomiting and no use of rescue antiemetics within 24 h post-surgery. Secondary endpoints included: the PONV rate, complete control (CC) rate (defined as no vomiting or retching, no nausea, and no use of rescue antiemetics within 24 h post-surgery), as well as Simplified PONV Impact Scale scores, clinically important PONV rate (defined as a PONV event with a Simplified PONV Impact Scale score of ≥5), rescue antiemetic use rate and frequency, cumulative vomiting frequency, the vomit and nausea subscale scores of the Simplified PONV Impact Scale, and the nausea visual analog scale (VAS) score within 24 h post-surgery. Additionally, clinicians, pharmacists, and nurses assessed treatment-related adverse events (AEs) from the initiation of the antiemetic prophylaxis regimen until 24 h postoperatively. This assessment included physical examination and laboratory results.

### 2.7 Statistical analysis

Continuous data were presented as mean ± standard deviation (SD), while categorical data were expressed as frequencies and percentages. Statistical analyses included Student’s t-test, Mann–Whitney test, Chi-square test, or Wilcoxon Signed Rank test, depending on the type and distribution of the data. To address potential selection bias due to the lack of random assignment, we conducted a propensity score-adjusted analyses using inverse probability of treatment weighting (IPTW). IPTW was employed to adjust for confounding factors such as Apfel score, ASA-PS, anesthesia duration, anesthesia type, dexmedetomidine, total infusion volume, crystalloid solution volume, and surgical type. Subsequently, sensitivity analyses were performed using univariate logistic regression, multivariate logistic regression models, and IPTW-adjusted logistic regression to investigate the association between aprepitant-based prophylaxis and composite PONV endpoints within 24 h after MBS. The multivariate logistic regression model included covariates with *p* < 0.05 from the univariate analysis, as well as variables previously identified in the literature as affecting outcome events. Specifically, the multivariable model adjusted for sex, surgical type, operative duration, aprepitant use, smoking history, motion sickness history, type 2 diabetes mellitus (T2DM), anesthesia duration, anesthesia type, total infusion volume, and crystalloid solution volume. No imputation was performed for missing data. Statistical significance was defined as two-tailed *p* < 0.05. All analyses were conducted using R statistical software (version 4.1.3) and SPSS (version 27.0; IBM Corp., Armonk, NY, United States).

### 2.8 Ethical approval and informed consent

This study protocol was approved by the Clinical Research Ethics Committee of Nanjing Drum Tower Hospital (2023–607-02) and was strict conducted in accordance with the Declaration of Helsinki Declaration. Since this study is based on a retrospective analysis of pre-existing PONV database data, the individual informed consent of participants was waived from the ethics committee.

## 3 Results

### 3.1 Patient characteristics

**Figure 1:**
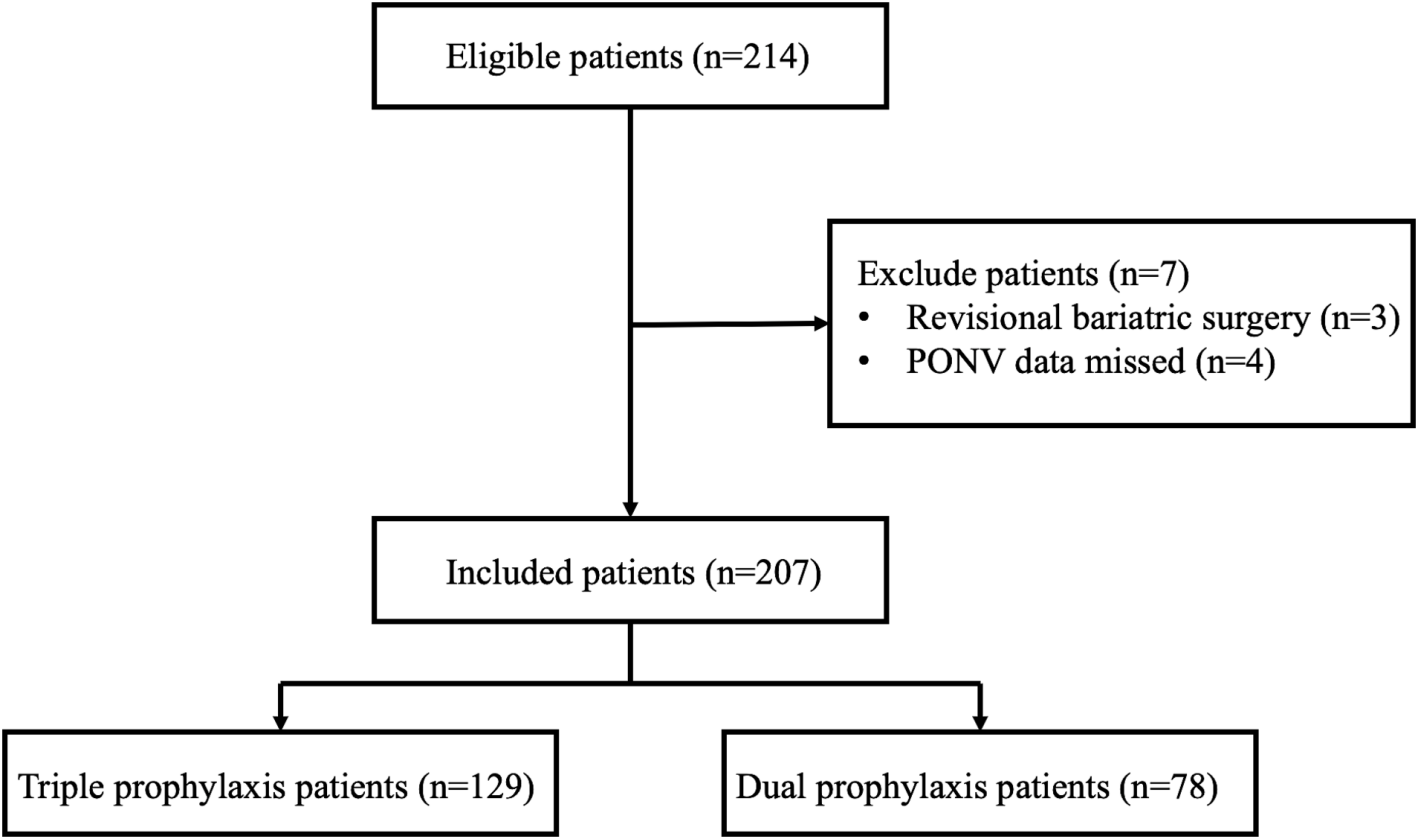
Flow diagram. PONV, Postoperative nausea and vomiting; Dual prophylaxis: ondansetron 8 mg and dexamethasone 10 mg; Triple prophylaxis: ondansetron 8 mg, dexamethasone 10 mg and aprepitant 125 mg.

A total of 214 metabolic bariatric surgeries were performed by the Pancreatic and Metabolic Surgery Department at Nanjing Drum Tower Hospital Affiliated to Nanjing University Medical School between December 28, 2023, and May 31, 2024. Ultimately, 207 patients were included in the study (Figure 1). Among these 207 patients, 156 (75.4%) experienced PONV, 112 (54.1%) had postoperative vomiting or retching (POV), and 133 (64.3%) had postoperative nausea (PON). There were 35 cases (16.9%) of clinically important PONV events, and 54 patients (26.1%) received rescue antiemetic treatment. All 207 patients received the standard dual antiemetic prophylaxis (8 mg ondansetron combined with 10 mg dexamethasone), and 78 patients receiving additional aprepitant prophylaxis. Statistical differences were observed between the two groups in terms of ASA-PS, anesthesia duration, anesthesia methods, and crystalloid solution volume. No significant differences were found in other demographic characteristics, anesthesia, or surgical characteristics (Table 1).

**Table 1:** Demographic and procedure characteristics of the study cohort before and after IPTW.

	Unmatched cohort			IPTW cohort		
Characteristic	Dual prophylaxis	Triple prophylaxis	*p* value	Dual prophylaxis	Triple prophylaxis	*p*^†^ value
*n*	129	78		216.1	186.8	
Demographics						
Age, mean (SD), year	32.0(10.8)	29.9 (7.3)	0.122	30.9 (10.7)	29.7 (7.4)	0.446
Female, *n* (%)	77 (59.7)	46 (59.0)	0.919	127.0 (58.8)	106.5 (57.0)	0.846
BMI, mean (SD), kg/m^2^	39.96 (6.47)	40.65 (7.68)	0.487	39.91 (6.25)	40.25 (7.17)	0.757
Comorbidities, *n* (%)						
Hypertension	31 (24.0)	21 (26.9)	0.921	45.2 (20.9)	53.7 (28.7)	0.293
T2DM	29 (22.5)	18 (23.1)	0.642	42.9 (19.9)	44.0 (23.5)	0.603
HbA1c, mean (SD), %	6.06 (1.39)	6.13 (1.29)	0.701	6.08 (1.34)	6.17 (1.28)	0.692
Smoking history, *n* (%)	35 (27.1)	24 (28.5)	0.574	158.6 (73.4)	132.3 (70.8)	0.739
Motion sickness, *n* (%)	32 (24.8)	16 (23.2)	0.478	45.1 (20.9)	45.3 (24.3)	0.655
Apfel risk score			0.323			0.980
0	18 (14.0)	13 (16.7)		33.5 (15.5)	34.1 (18.3)	
1	37 (28.7)	20 (25.6)		53.6 (24.8)	43.6 (23.3)	
2	54 (41.9)	39 (50.0)		103.8 (48.0)	87.0 (46.5)	
3	20 (15.5)	6 (12.6)		25.1 (11.6)	22.2 (11.9)	
ASA-PS, *n* (%)			0.021*			0.818
I	8 (6.2)	1 (1.3)		9.1 (4.2)	11.3 (6.1)	
II	56 (43.4)	48 (61.5)		103.0 (47.7)	95.9 (51.3)	
III	65 (50.4)	29 (37.2)		104.0 (48.1)	79.6 (42.6)	
Anesthesia						
Anesthesia duration, mean (SD), min	73.38 (21.35)	67.15 (20.31)	0.040	71.45 (19.53)	69.90 (21.29)	0.658
Anesthesia methods, *n* (%)			<0.001			0.799
TIVA	41 (31.8)	57 (73.1)		108.5 (50.2)	98.2 (52.6)	
Combined intravenous and inhalation anesthesia	88 (68.2)	21 (26.9)		107.6 (49.8)	88.6 (47.4)	
Intraoperative medications						
Fentanyl, *n* (%)	103 (79.8)	59 (75.6)	0.477	151.0 (69.9)	131.1 (70.2)	0.973
Sufentanil, *n* (%)	26 (20.2)	20 (25.6)	0.358	65.1 (30.1)	57.4 (30.7)	0.951
Dexmedetomidine, *n* (%)	123 (95.3)	69 (88.5)	0.064	197.6 (91.5)	170.6 (91.3)	0.975
Refentanil, mean (SD), μg	764.65 (391.11)	702.03 (374.54)	0.258	717.55 (365.29)	784.14 (406.07)	0.327
Refentanil, mean (SD), μg/kg	6.97 (3.99)	6.14 (3.26)	0.122	6.51 (3.68)	6.94 (3.58)	0.499
Propofol, mean (SD), mg	558.31 (159.54)	554.28 (229.12)	0.882	544.03 (158.73)	626.84 (276.34)	0.089
Propofol, mean (SD), mg/kg	5.00 (1.67)	4.78 (1.87)	0.390	4.87 (1.65)	5.41 (2.10)	0.151
Urine volume, mean (SD), l	329.07 (199.87)	348.72 (276.69)	0.555	336.35 (191.17)	365.59 (296.33)	0.522
Total infusion volume, mean (SD), l	1644.57 (409.80)	1753.85 (387.28)	0.059	1722.76 (445.44)	1739.86 (402.58)	0.837
Crystalloid solution volume, mean (SD), l	887.98 (324.32)	1048.72 (313.96)	0.001	983.19 (413.09)	1001.79 (274.87)	0.815
Colloid solution volume, mean (SD), l	755.81 (258.57)	701.28 (269.92)	0.150	739.02 (255.44)	735.79 (276.83)	0.945
Operation						
Operative duration, mean (SD), min	59.84 (20.68)	54.45 (20.29)	0.068	58.21 (19.00)	57.19 (21.31)	0.773
Surgical type, *n* (%)			0.06			0.815
SG	85 (65.9)	61 (78.2)		152.4 (70.5)	135.4 (72.5)	
SG-SASJ/I	44 (34.1)	17 (21.8)		63.7 (29.5)	51.5 (27.5)	
Bleeding volume, mean (SD), ml	34.22 (18.05)	32.05 (16.31)	0.385	32.01 (18.18)	31.36 (15.62)	0.826

IPTW, Inverse probability of treatment weighting; ASA-PS, American Society of Anesthesiologists–Physical Status; BMI, Body mass index; T2DM, Type 2 diabetes mellitus; HbA1c, Glycated Hemoglobin A1c; TIVA, Total intravenous anesthesia; SG, Sleeve Gastrectomy; SASJ/I, Single anastomosis sleeve jejunal/ileal bypass; SD, Standard deviation; IQR, Interquartile Range; Statistical tests used were: t-test or Mann–Whitney U test for continuous variables, Chi-square test for categorical variables, and Wilcoxon rank sum test for ordinal data; IPTW was used to adjust for confounding by Apfel score, ASA-PS, anesthesia duration, anesthesia type, dexmedetomidine, total infusion volume, crystalloid solution volume, and surgical type.†p- values are IPTW-adjusted.

### 3.2 Effectiveness of aprepitant-based triple prophylaxis in reducing PONV within 24 h post-metabolic bariatric surgery

The triple prophylactic regimen containing aprepitant showed a significantly lower incidence of PONV compared to the dual prophylactic regimen without aprepitant (59.0% vs. 85.3%, *p* < 0.001) (Table 2). It also resulted in a lower rate of rescue antiemetics (7.7% vs. 37.2%, *p* < 0.001), reduced frequency of rescue antiemetics (*p* < 0.001), and a lower score on the simplified PONV Impact Scale (*p* < 0.001; Table 2). Additionally, the triple regimen achieved a higher CR rate (82.1% vs. 24.0%, *p* < 0.001) and a higher CC rate (41.0% vs. 14.7%, *p* < 0.001; Table 2). The IPTW-adjusted analysis further confirmed the robustness of these findings. Notably, the IPTW-adjusted analysis did not reveal a significant difference between the two groups in terms of clinically important PONV rate (Table 2). Univariate and multivariate logistic regression analyses, as well as inverse probability of treatment weighting (IPTW)-adjusted logistic regression, demonstrated that aprepitant-containing prophylaxis significantly reduced the incidence of PONV, rescue antiemetics rate, and complete response failure rate within 24 h post-MBS (Table 3). However, the effect of aprepitant on clinically significant PONV rate was less consistent, with IPTW-adjusted analysis not showing a significant reduction (Table 3).

**Table 2:** Comparison of composite PONV endpoints within 24 h after metabolic bariatric surgery with and without aprepitant.

	Study cohort (*N* = 207)			
Outcome	Dual prophylaxis (*n* = 129)	Triple prophylaxis (*n* = 78)	*p* value	*p*^†^ value
PONV, *n* (%)	110 (85.3)	46 (59.0)	<0.001	0.003
Simplified PONV Impact Scale score, *n* (%)			<0.001	<0.001
0	19 (14.7)	32 (41.0)		
1	11 (8.5)	26 (33.3)		
2	10 (7.8)	8 (10.3)		
3	20 (15.5)	6 (7.7)		
4	37 (28.7)	3 (3.8)		
5	21 (16.3)	3 (3.8)		
6	11 (8.5)	0 (0)		
Clinically important PONV, *n* (%)	32 (24.8)	3 (3.8)	<0.001	0.095
Rescue antiemetics, *n* (%)	48 (37.2)	6 (7.7)	<0.001	<0.001
Frequency of rescue antiemetics, *n* (%)			<0.001	0.003
0	81 (62.8)	71 (91.0)		
1	32 (24.8)	5 (6.4)		
2	12 (9.3)	2 (2.6)		
3	4 (3.1)	0 (0.0)		
Complete response rate, *n* (%)	31 (24.0)	64 (82.1)	<0.001	<0.001
Complete control rate, *n* (%)	19 (14.7)	32 (41.0)	<0.001	0.003
Complete control failure rate, *n* (%)	98 (76.0)	14 (17.9)	<0.001	<0.001

PONV, Postoperative nausea and vomiting; Complete response was defined as no vomiting and no use of rescue antiemetics within 48 h postoperatively; Complete control was defined as no nausea and vomiting, and no use of rescue antiemetics within 48 h postoperatively; Statistical tests used were: Chi-square test for categorical variables, and Wilcoxon rank sum test for ordinal data; IPTW was used to adjust for confounding by Apfel score, ASA-PS, anesthesia duration, anesthesia type, dexmedetomidine, total infusion volume, crystalloid solution volume, and surgical type. †*p*- values are IPTW-adjusted.

**Table 3:** Associations between aprepitant and composite endpoints of PONV after metabolic bariatric surgery in the crude analysis, multivariable analysis, IPTW-adjusted analysis.

	Univariable analysis		Multivariable analysis		IPTW-adjusted analysis	
	Crude OR (95% CI)	*p* value	adjusted OR (95% CI)	*p* value	adjusted OR (95% CI)	*p* value
Complete response rate, *n* (%)	14.452 (7.325, 30.210)	<0.001	28.623 (10.847, 86.998)	<0.001	10.312 (4.186, 25.399)	<0.001
PONV, *n* (%)	0.248 (0.128, 0.482)	<0.001	0.222 (0.090, 0.519)	<0.001	0.287 (0.125, 0.663)	0.004
Clinically important PONV, *n* (%)	0.121 (0.036, 0.411)	<0.001	0.063 (0.011, 0.249)	<0.001	0.270 (0.054, 1.350)	0.11
Rescue antiemetics, *n* (%)	0.141 (0.057, 0.348)	<0.001	0.105 (0.033, 0.286)	<0.001	0.155 (0.052, 0.457)	<0.001
Complete response failure rate, *n* (%)	0.069 (0.034, 0.140)	<0.001	0.035 (0.011, 0.092)	<0.001	0.097 (0.039, 0.239)	<0.001

IPTW, Inverse probability of treatment weighting, OR, Odd ratio; CI, Confidence interval; Univariable, multivariable, and IPTW-adjusted logistic regression analyses were used to evaluate an antiemetic efficacy associated with composite endpoints of PONV; IPTW was used to adjust for confounding by Apfel score, ASA-PS, anesthesia duration, anesthesia type, dexmedetomidine, total infusion volume, crystalloid solution volume, and surgical type; The multivariable model adjusted for sex, surgical type, operative duration, aprepitant, smoking history, motion sickness history, T2DM, anesthesia duration, anesthesia type, total infusion volume, and crystalloid solution volume.

### 3.3 Impact of aprepitant-based triple prophylaxis on POV and PON

For POV, the aprepitant-containing prophylactic regimen significantly reduced the incidence of POV, vomiting frequency, and vomiting subscale scores (*p* < 0.001; Table 4). For PON, Although IPTW-adjusted analysis did not show a statistically significant difference in PON rates between the two groups, the aprepitant group had significantly lower Nausea VAS score and Nausea Subscale score (*p* < 0.05; Table 4). These findings suggest that the aprepitant-containing regimen can effectively reduce both the severity and frequency of PON.

**Table 4:** Comparison of POV and PON endpoints within 24 h after metabolic bariatric surgery with and without aprepitant.

	Study cohort (*N* = 207)			
Outcome	Dual prophylaxis (*n* = 129)	Triple prophylaxis (*n* = 78)	*p* value	*p*^†^ value
Vomiting, *n* (%)	98 (76.0)	14 (17.9)	<0.001	<0.001
Vomiting episode, Median [IQR]	5 [1, 10]	0 [0, 0]	<0.001	<0.001
Vomiting Subscale score, *n* (%)			<0.001	<0.001
0	31 (24.0)	64 (82.1)		
1	6 (4.7)	1 (1.3)		
2	10 (7.8)	4 (5.1)		
3	82 (63.6)	9 (11.5)		
Nausea, *n* (%)	92 (71.3)	41 (52.6)	0.006	0.109
Nausea VAS score, Median [IQR]	3.0 [0.0, 5.0]	2.0 [0.0, 3.0]	<0.001	0.009
Nausea Subscale score, *n* (%)			0.001	0.035
0	37 (28.7)	37 (47.4)		
1	58 (45.0)	31 (39.7)		
2	21 (16.3)	10 (12.8)		
3	13 (10.1)	0 (0)		

POV, Postoperative vomiting; PON, Postoperative nausea; Statistical tests used were: Chi-square test for categorical variables, and Wilcoxon rank sum test for ordinal data; IPTW was used to adjust for confounding by Apfel score, ASA-PS, anesthesia duration, anesthesia type, dexmedetomidine, total infusion volume, crystalloid solution volume, and surgical type; ^†^*p*- values are IPTW-adjusted.

### 3.4 Safety evaluation

The most common adverse effect in both groups was constipation, with a rate of 16.3% in the aprepitant prophylaxis group and 17.9% in the control group; a minority of these cases were classified as CTCAE Grade 2 (Table 5). Other adverse events, such as fatigue, diarrhea, hiccups, increased ALT, and increased AST, all occurred at rates below 10% and were classified as CTCAE Grade 1 (Table 5). Due to the interference of surgical and anesthetic factors, adverse events such as abdominal pain, headache, dizziness, appetite loss, and insomnia were not assessed in this study. No serious adverse events were reported among the 207 patients.

**Table 5:** Treatment-related adverse events.

	Dual prophylaxis (*n* = 129)		Triple prophylaxis (*n* = 78)		*p* value
Adverse events	Grade 1	Grade 2	Grade 1	Grade 2	
Fatigue, *n* (%)	12 (9.3)	0 (0)	6 (7.7)	0 (0)	0.691
Constipation, *n* (%)	16 (12.4)	5 (3.9)	11 (14.1)	3 (3.8)	0.976
Diarrhea, *n* (%)	4 (3.1)	0 (0)	7 (9.0)	0 (0)	0.069
Increased ALT, *n* (%)	8 (6.3)	0 (0)	6 (7.7)	0 (0)	0.680
Increased AST, *n* (%)	7 (5.4)	0 (0)	5 (6.4)	0 (0)	0.770
Hiccups, *n* (%)	3 (2.3)	0 (0)	1 (1.3)	0 (0)	0.598

ASL, Aspartate aminotransferase; ALT, Alanine aminotransferase; Graded according to the Common Terminology Criteria for Adverse Events (CTCAE); Statistical tests used were Wilcoxon rank sum test.

## 4 Discussion

There is a paucity of data regarding the efficacy of a combined prophylactic regimen of aprepitant (125 mg), ondansetron, and dexamethasone in preventing PONV in MBS patients. The current study aimed to evaluate whether the addition of a single preoperative dose of aprepitant (125 mg) to a standard dual antiemetic regimen could further improve outcomes related to composite PONV endpoints. Propensity score matching was employed to mitigate selection bias. Our results demonstrated that the addition of a single dose of aprepitant to a standard dual antiemetic regimen was associated with a further reduction in the incidence of PONV, the frequency and incidence POV, rescue antiemetic use, simplified PONV impact scale scores, nausea VAS scores and nausea subscale scores 24 h after MBS. Furthermore, the CR rate and CC rate were significantly higher in the aprepitant**-**based triple prophylaxis group. Univariate, multivariate, and propensity score-weighted logistic regression analyses consistently supported these findings. Importantly, the safety profile of the aprepitant group was comparable to the control group, with a low incidence of adverse events, most of which were mild (CTCAE grade 1). It is worth noting that this study is the first to investigate the use of a single 125 mg dose of aprepitant in a multimodal antiemetic regimen for MBS.

In line with our results, previous studies have evaluated the efficacy of aprepitant as a single-agent add-on to multimodal antiemetic regimens in reducing PONV following MBS (13–17). In a pioneering study by Ashish C Sinha et al., the first to examine the role of aprepitant in a multimodal antiemetic regimen for MBS, a randomized controlled trial demonstrated that the combination of aprepitant (80 mg) and ondansetron (4 mg) significantly reduced the cumulative incidence of vomiting at 72 h postoperatively (3% vs. 15%, *p* = 0.021) compared to ondansetron (4 mg) alone. Additionally, this combination resulted in a higher CC rate of PONV (42.18% vs. 36.67%) and a significant delay in time to first emesis (*p* = 0.019) (15). In a double-blind randomized controlled trial by Elías Ortiz et al., patients who received a standard triple antiemetic regimen plus prophylactic oral aprepitant 80 mg 1 h preoperatively experienced significantly lower rates of PONV at various postoperative time points compared to those who received the standard triple regimen alone (dexamethasone 10 mg, ondansetron 4 mg, and metoclopramide 10 mg). Specifically, the combined regimen demonstrated a lower incidence of PONV at early postoperative, 6-h, 12-h, and 24-h assessments (51.7% vs. 34.3, 65.8% vs. 33.3, 57.8% vs. 20.4, and 33.7% vs. 10.9%, respectively, *p* ≤ 0.001). Furthermore, the aprepitant-augmented group exhibited lower rates of postoperative vomiting and rescue antiemetic medication use (10). Tarek M. Ashoor et al. conducted a randomized controlled trial comparing the efficacy of three antiemetic regimens in preventing PONV following LSG. The first regimen combined aprepitant (80 mg) and dexamethasone (8 mg), while the second combined mirtazapine (30 mg) and dexamethasone (8 mg). Both dual regimens demonstrated significantly higher complete response rates compared to the single-agent dexamethasone (8 mg) group (79.3 and 78.6% vs. 20.7%, respectively) (13). Therneau et al. conducted a retrospective study demonstrating that the addition of aprepitant (40 mg) to a standard triple antiemetic regimen (dexamethasone, droperidol, and ondansetron) significantly decreased PONV incidence in the postanesthesia care unit (PACU) and within the first hour post-PACU. However, there was no statistically significant difference in the overall incidence of PONV at 48 h postoperatively between the two groups (16). Zhu et al.’s meta-analysis on the efficacy of aprepitant in preventing post-MBS nausea and vomiting suggested that aprepitant only reduced PONV incidence at 0, 6, and 12 h postoperatively, without affecting PONV incidence at 24 or 48 h postoperatively (17). This seems reasonable given aprepitant’s half-life of 9–12 h. However, this conclusion may not be entirely accurate as it is not based on consistent findings across multiple studies. We believe that the dosage of aprepitant may play a significant role. Studies demonstrating no difference in PONV incidence at 24 or 48 h predominantly used a single 40 mg dose of aprepitant, whereas studies employing a single 80 mg dose of aprepitant showed a significant reduction in PONV incidence at 24 h. Similarly, our study using a single 125 mg dose of aprepitant also significantly reduced PONV incidence. Therefore, we propose that a single 80–125 mg dose may be more appropriate for MBS patients.

A consistent body of evidence supports the efficacy of aprepitant, either as a standalone treatment or as part of a combination therapy, in achieving favorable complete response rates for nausea and vomiting. To date, published studies evaluating the efficacy of single-dose aprepitant in MBS have primarily focused on 40 and 80 mg doses. Our study is the first to report the efficacy of a single 125 mg dose of aprepitant in preventing PONV in MBS, achieving CR and CC rates of 82.1 and 41.0%, respectively. These findings are comparable to those reported by Ashish C Sinha, who observed a CC rate of 42.18% with a combination of aprepitant (80 mg) and ondansetron (4 mg), and Tarek M. Ashoor et al., who reported a CR rate of 79.3% with a combination of aprepitant (80 mg) and dexamethasone (8 mg). The observed variations in CR and CC rates across different studies may be attributed to factors such as aprepitant dosage, baseline characteristics of the study population, and risk factors for PONV. Given the high prevalence of obesity among patients undergoing MBS, our findings suggest that a single 125 mg dose of aprepitant may be a more appropriate option.

Consistent with previous findings (13–17), our study demonstrates that multimodal regimens incorporating aprepitant effectively reduce the incidence and frequency of postoperative vomiting in MBS patients. Elías Ortiz et al. reported that a standard triple antiemetic regimen augmented with prophylactic oral aprepitant significantly decreased nausea severity at various postoperative time points compared to the standard regimen alone. Our study similarly found that the triple regimen containing aprepitant significantly reduced postoperative 24-h nausea VAS scores and Nausea Subscale scores. Intriguingly, however, the incidence of postoperative nausea was not significantly reduced. These findings align with those of Sinha AC et al., who reported no significant improvement in postoperative nausea with aprepitant. These results collectively suggest that while aprepitant is effective in reducing vomiting, its efficacy in mitigating nausea remains uncertain and warrants further investigation. The selective suppression of vomiting without a concomitant reduction in nausea by aprepitant is an intriguing clinical phenomenon that may provide insights into the role of NK-1R in both central and peripheral neural pathways.

PONV typically peak within the first 4 h after surgery (18). Aprepitant, a selective neurokinin-1 (NK1) receptor antagonist, is a long-acting antiemetic with a half-life of 9–13 h and is highly effective in preventing opioid-induced emesis (10, 19–21). This suggests that a single dose of aprepitant could effectively cover the high-risk period for PONV, indicating its potential as a prophylactic agent for preventing vomiting in MBS patients. Multiple studies have demonstrated that the addition of a single dose of aprepitant significantly reduces the incidence and severity of early PONV. Ashish C Sinha et al. reported that the addition of a single dose of aprepitant significantly delayed the time to the first occurrence of PONV (15). Pharmacologically, aprepitant, unlike fluphenazine, mirtazapine, olanzapine, and promethazine, does not exhibit sedative effects, making it particularly suitable for use in people with obesity. Most studies evaluating aprepitant doses ranging from 40 to 125 mg have not reported significant adverse effects (22–27). While some studies have described adverse events such as dizziness, fatigue, hiccups, dehydration, diarrhea, and elevated liver function tests (28, 29), it is challenging to definitively attribute these events to aprepitant due to the confounding effects of anesthesia and surgery. In our study, adverse events were generally mild and well-tolerated in both groups, with no treatment-related serious adverse events reported, and no patient discontinued treatment due to adverse events. These findings suggest that a single 125 mg dose of aprepitant is safe for use in people with obesity undergoing MBS.

Alternative antiemetic strategies have also shown promise in managing PONV, particularly in patients undergoing MBS. Ebrahimian et al. demonstrated that a combination of metoclopramide and ondansetron, when implemented alongside Enhanced Recovery after Surgery (ERAS) protocols, significantly reduced PONV incidence compared to single-agent therapies or control groups and eliminated the need for rescue antiemetics (30). Similarly, Fathy et al. explored the novel approach of injecting a magnesium sulfate-lidocaine mixture into the pylorus during LSG, which led to a substantial reduction in gastric intraluminal pressure and significantly lowered PONV rates at both 6 and 24 h postoperatively (17.1% vs. 91.4 and 0% vs. 40%, respectively, *p* < 0.0001) (31). Additionally, Lam et al. reported that the perioperative use of low-dose haloperidol safely decreased PONV episodes and reduced hospital length of stay in bariatric patients following minimally invasive surgery, emphasizing its potential as part of a multimodal regimen (32). These studies collectively highlight the critical importance of multimodal and multi-agent antiemetic strategies in managing PONV, particularly in high-risk bariatric populations. The evidence underscores that exploring and implementing various multimodal prophylactic regimens can significantly enhance the prevention of PONV in the postoperative period. Approaches such as the combination of metoclopramide and ondansetron, perioperative administration of low-dose haloperidol, or innovative interventions like pyloric injections with magnesium sulfate-lidocaine mixtures offer promising alternatives. When integrated with established therapies, such as aprepitant-based regimens, these strategies contribute to a more comprehensive and tailored approach to PONV prophylaxis, facilitating optimized outcomes for bariatric surgery patients. Further research on the efficacy of diverse multimodal approaches is essential to refine and advance PONV prevention protocols in this vulnerable cohort.

This study has several limitations that warrant careful consideration. First, its retrospective and observational design introduces selection bias, as it relies on previously collected data that may not accurately represent the broader population. Inclusion criteria may differ from those of a prospective study. Second, the retrospective nature of the study is susceptible to recall bias, and the data used may be incomplete or inconsistent, potentially affecting the reliability of the results. Third, retrospective observational studies cannot establish causation and are subject to confounding factors. Fourth, the lack of randomization and the significant disparity in sample sizes introduced imbalances between the two groups, which were mitigated to some extent by propensity score matching analysis. However, the potential for residual confounding from unmeasured variables remains a limitation of this observational study. Fifth, the single-center nature of this study limits the generalizability of our findings to broader populations or different institutional settings. Additionally, the limited sample size precluded subgroup analyses based on specific surgical procedures, which might have provided further insights into the differential effects of our intervention. To address these limitations and strengthen the validity of our findings, larger, multicenter studies are needed to enhance the external validity of the results. Furthermore, we plan to conduct a randomized controlled trial to rigorously test our hypothesis and provide higher-quality evidence to guide clinical practice.

## 5 Conclusion

Our findings indicate that the addition of a single preoperative dose of aprepitant to a dual antiemetic prophylaxis of dexamethasone and ondansetron might be associated with a further improve outcomes related to composite PONV endpoints in patients undergoing metabolic bariatric surgery.

## Data Availability

The original contributions presented in the study are included in the article/supplementary material, further inquiries can be directed to the corresponding author/s.
